# Effects of CD36 Genotype on Oral Perception of Oleic Acid Supplemented Safflower Oil Emulsions in Two Ethnic Groups: A Preliminary Study

**DOI:** 10.1111/1750-3841.14115

**Published:** 2018-04-16

**Authors:** Brenda Burgess, Melania Melis, Katelyn Scoular, Michael Driver, Karen M. Schaich, Kathleen L. Keller, Iole Tomassini Barbarossa, Beverly J. Tepper

**Affiliations:** ^1^ Dept. of Food Science and Center for Sensory Sciences & Innovation, School of Environmental and Biological Sciences Rutgers Univ. New Brunswick N.J. U.S.A; ^2^ Dept. of Biomedical Sciences, Section of Physiology Univ. of Cagliari Monserrato Italy; ^3^ Dept. of Nutritional Sciences and Dept. of Food Science The Pennsylvania State Univ., University Park Pa. U.S.A

**Keywords:** CD36, fatty acid, fat taste, oleic acid, safflower oil

## Abstract

Previous studies demonstrate humans can detect fatty acids via specialized sensors on the tongue, such as the CD36 receptor. Genetic variation at the common single nucleotide polymorphism rs1761667 of *CD36* has been shown to differentially impact the perception of fatty acids, but comparative data among different ethnic groups are lacking. In a small cohort of Caucasian and East Asian young adults, we investigated if: (1) participants could detect oleic acid (C18:1) added to safflower oil emulsions at a constant ratio of 3% (w/v); (2) supplementation of oleic acid to safflower oil emulsions enhanced perception of fattiness and creaminess; and (3) variation at rs1761667 influenced oleic acid detection and fat taste perception. In a 3‐alternate forced choice test, 62% of participants detected 2.9 ± 0.7 mM oleic acid (or 0.08% w/v) in a 2.8% safflower oil emulsion. Supplementation of oleic acid did not enhance fattiness and creaminess perception for the cohort as a whole, though East Asians carrying the GG genotype perceived more overall fattiness and creaminess than their AA genotype counterparts (*P* < 0.001). No differences were observed for the Caucasians. These preliminary findings indicate that free oleic acid can be detected in an oil‐in‐water emulsion at concentrations found in commercial oils, but it does not increase fattiness or creaminess perception. Additionally, variation at rs1761667 may have ethnic‐specific effects on fat taste perception.

## Introduction

The five basic tastes of sweet, salty, sour, bitter, and umami are detected on the tongue via stimulation of specialized receptors. It was originally thought that dietary fats had no “taste” of their own, but rather were sensed through flavor and textural cues. This view was based on earlier evidence suggesting that humans do not produce sufficient amounts of lingual lipase (Schiffman, Graham, Sattely‐Miller, & Warwick, 1998; Spielman, D'Abundo, Field, & Schmale, 1993) to cleave triglycerides into fatty acids for activation of receptors in the mouth. Emerging discoveries have since challenged this understanding by demonstrating that fatty acids are liberated during oral processing and can be detected orally when all other sensory cues are minimized. Specifically, Chale‐Rush, Burgess, and Mattes (2007a, 2007b) and Stewart, Newman, and Keast (2011) showed human assessors could detect fatty acids in liquid emulsions thickened with gums to mimic the mouthfeel of fat. Furthermore, thresholds were found to vary by fatty acid chain length and degree of saturation (Chale‐Rush et al., 2007a, 2007b; Stewart et al., 2010). The term “oleogustus” has been coined by Running, Craig, and Mattes (2015) to describe the unique oral sensations elicited by free fatty acids that are distinct from the positive “creamy” and “fatty” attributes associated with dietary fats.

Various classes of fatty acid receptors are expressed in multiple cell types and tissues (Hajri & Abumrad, 2002; Silverstein & Febbraio, 2009; Su & Abumrad, 2009), including the lingual epithelium (Simons, Kummer, Luiken, & Boon, 2011). CD36, a scavenger protein that binds a wide array of lipids including oxidized lipoproteins, phospholipids, and cholesterol (Febbraio, Hajjar, & Silverstein, 2001; Silverstein, Li, Park, & Rahaman, 2010), is primarily responsible for detection of long chain fatty acids on the tongue (Ozdener et al., 2014; Reed & Xia, 2015). Production of this protein is controlled by the *CD36* gene and regulated by variations within its genetic coding (Love‐Gregory et al., 2011). In particular, the substitution of A for G in the rs1761667 single nucleotide polymorphism (SNP) has been shown to decrease expression of this protein (Love‐Gregory et al., 2011) and associate with a reduced ability to detect fatty acids orally (Melis, Sollai, Muroni, Crnjar, & Barbarossa, 2015; Mrizak et al., 2015; Pepino, Love‐Gregory, Klein, & Abumrad, 2012). Recent evidence also suggests that this polymorphism is differentially associated with plasma lipid markers, such as endocannabinoid levels, and body composition in lean and obese individuals (Melis et al., 2017).

Presumably, if SNPs in CD36 play a substantial role in altering oral fat perception, then the presence of such alleles could heighten the risk of excess fat consumption, which may lead to weight gain. Previous studies examining fat taste responsiveness by CD36 genotype have been conducted predominantly in overweight/obese cohorts of African Americans (Keller et al., 2012; Pepino, Finkbeiner, Beauchamp, & Mennella, 2010), Tunisian women (Mrizak et al., 2015), or Malaysians (Ong, Tan, & Say, 2017). Only one investigation in Italy assessed individuals with healthy body weights (Melis et al., 2015). Thus, studies evaluating the impact of CD36 SNPs on orosensory perceptions among lean individuals of different ethnic groups are sparse. This preliminary study was designed, in part, to address these gaps in the literature.

This study was also designed to investigate new approaches in fat reduction. For the past several decades, the food industry has directed its efforts toward reducing or eliminating the amount of fat in packaged foods through the use of modified lipids (for example, Olestra and Salatrim) or carbohydrate‐ and protein‐based derivatives (see review: McClements, 2015). Fat contributes to a food's flavor profile and appearance, increases nutrient absorption, stimulates hormone release and slows gastric emptying, so finding suitable fat substitutes that possess these sensory and biological properties has been challenging (McClements, 2015). Another potential approach might be to reduce the fat content of a food, but increase its mouthfeel (for example, fattiness, creaminess) using naturally occurring fatty acids. This strategy could be feasible since commercial fats such as safflower, olive, and palm oils naturally contain 0.5% to 5.0% free fatty acids (by weight) (Aydeniz, Güneşer, & Yılmaz, 2014; Che Man, Moh, & van de Voort, 1999; El‐Abassy, Donfack, & Materny, 2009). Thus, supplementing a food with a complementary free fatty acid might be an avenue toward enhancing fattiness and creaminess perception.

This study had several objectives. We first aimed to determine if naïve human assessors could detect free oleic acid added to safflower oil emulsions and whether this supplementation influenced perceived fattiness and creaminess. We then investigated if variations in the rs1761667 SNP of CD36 moderated detection of the fatty acids and perception of fat content. Finally, we examined if these oral responses to fat differed between participants who self‐identified as Caucasian and East Asian.

## Methods

### Participants

Participants were screened from a convenience sample of students and staff at Rutgers Univ. At the Sensory Evaluation/Nutrition Laboratory, individuals had their height and weight measured, and they completed a health history form and demographic questionnaire. They self‐reported their ethnic identity by checking a box on the demographic questionnaire (Caucasian, East Asian, Pacific Islander, and so on) and they also reported their country of birth. We classified individuals as “Caucasian” if they self‐identified as Caucasian and were born and/or raised in the United States. We classified Individuals as “East Asian” if they self‐identified as East Asian, and were born and/or raised in China, Taiwan, or Korea. Individuals who indicated a mixed identity (for example, Caucasian and Asian) or those born and/or raised in other Asian countries (for example, India) were disqualified in order to promote homogeneity within the two population groups of interest. Since we did not conduct genetic ancestry testing, the precise ancestry of each participant could not be ascertained. The Institutional Review Board at Rutgers approved the study and all participants provided written, informed consent prior to engaging in the research activities. Compensation was given after the final test session.

### Assessment of CD36 status

Cells were collected from each participant by gently rolling a swab (Epicentre, Madison, WI, U.S.A.) on the buccal surfaces of the mouth between the cheek and gums. DNA was extracted and purified using the Maxwell 16 Buccal Swab LEV DNA Purification Kit and the Promega Maxwell (Promega, Madison, WI, U.S.A.). The target area of CD36 (NCBI Gene Identity 948) was amplified using PCR primers (Forward: tccattgaagcccttctgtt, Reverse: attctaaggcgggaagcttc, Invitrogen, Carlsbad, CA, U.S.A.) and sequenced using the forward primer (High Throughput Genomics Center, Seattle, Wash., U.S.A.). Sequences were analyzed using the program Geneious (Geneious, Newark, NJ, U.S.A.) to determine the nucleic acid at position 13436 (SNP rs1761667, NCBI Reference Sequence NG_008192.1).

### Sample preparation

Refined, bleached and deodorized safflower oil (California Oil Corporation, Richmond, CA, U.S.A.) was chosen as the base of the samples because of its neutral taste and low susceptibility to oxidation (Fuller, Diamond, & Applewhite, 1967). Free oleic acid was used as the supplementary fatty acid because it occurs naturally in safflower oil (Orsavova, Misurcova, Ambrozova, Vicha, & Mlcek, 2015) and its gustatory detection threshold in aqueous solution has been previously described (Chale‐Rush et al., 2007b). To prepare the base emulsions, different concentrations of safflower oil (California Oil Corporation) ranging from 0.09% to 15.81% (w/v) (separated by quarter log steps) were added to a mixture of spring water, 10% (w/v) gum acacia (TIC gums, Belcamp, MD, U.S.A.), and 0.01% (w/v) EDTA (Sigma Aldrich, St. Louis, MO, U.S.A.). These safflower oil concentrations (Oleic–) were then homogenized at 7500 RPMs in chilled bottles for 5 min on crushed ice using a Polytron 1600 E high shear mixer (Kinematica, Inc., Bohemia, NY, U.S.A.). An identical set of emulsions (Oleic+) was prepared with free oleic acid (Sigma Aldrich) added at 3% (w/v) to the safflower oil, yielding concentrations ranging from 0.0027% to 0.4743% (w/v). Once homogenization was complete, aluminum foil was wrapped around the bottles and they were held on ice to prevent degradation from light and heat, respectively. All samples were prepared the morning of testing and held no longer than 5 hr. The samples were allowed to come to room temperature for 10 min before they were served. The composition of the samples can be found in Table 1.

**Table 1:** Composition of safflower oil emulsions for sensory testing.^a^^,^ b

	Control (–Oleic acid)	Supplemented (+Oleic acid)	
Sample number	Concentration of safflower oil (in % w/v)	Concentration of oleic acid (in % w/v)	Concentration of oleic acid (in mM)
1	0.09	0.003	0.09
2	0.16	0.005	0.17
3	0.28	0.008	0.30
4	0.50	0.015	0.53
5	0.89	0.027	0.94
6	1.58	0.047	1.68
7	2.81	0.084	2.99
8	5.00	0.150	5.31
9	8.89	0.267	9.44
10	15.81	0.474	16.79

^a^Each numbered sample contained safflower oil at the concentration indicated; all supplemented (Oleic acid+) samples also contained oleic acid at the concentrations indicated (at a constant ratio of 3% of the oil).

^b^Samples in the shaded rows were used in the attribute rating tests.

### Testing of lipid oxidation

All lipid materials were aliquoted and stored in a freezer (–18 °C) in acid‐washed glassware under argon gas to protect against oxidation. Upon being mixed with spring water, gum acacia, and EDTA, a liquid–liquid extraction using chloroform was performed on the lipid layer. This portion was screened for the presence of peroxides using the SafTest (MP Biomedical, Solon, OH, U.S.A.) and conjugated dienes using a Micro Chem II spectrophotometer (BBI Source Scientific, Garden Grove, CA, U.S.A.) and UV spectrophotometer (Cary 50 Bio, Varian, Palo Alto, CA, U.S.A.), respectively. When read at 234 nm, the acceptable range for the presence of peroxides is 0.3 to 1.2 μM and for conjugated dienes is 0.05 to 0.5 μM. Only trace amounts of either species were found in the lipid ingredients or the homogenized samples.

### Testing procedures

#### Test sessions

Testing was conducted in 2 sessions (detailed below), separated by at least 2 nontest days. Samples were served at room temperature and presented to participants in individual booths within the Sensory Evaluation/Nutrition Laboratory. Red lights were used to mask visual differences between the samples and nose clips (Speedo, London, U.K.) were worn to inhibit olfactory input (Bolton & Halpern, 2010). Participants were asked to refrain from consuming any food or beverages for 3 hr before each testing session.

#### Oleic acid threshold test

The 3‐alternative forced choice (3‐AFC) test was used to determine each participant's oleic acid detection threshold according to standard methods (ASTM E679‐04). Briefly, they received three clear, 2‐oz soufflé cups filled with 10 mL of an emulsion in a random arrangement generated by FIZZ software (Biosystèmes, version 2.47B, Couternon, France); two samples were unsupplemented (Oleic–) and the third was supplemented (Oleic+). Participants received instruction to rinse their mouths with spring water, then taste and expectorate each emulsion to identify the Oleic+ sample. Testing began at the lowest concentration of safflower oil and proceeded until participants correctly identified the Oleic+ sample three times in a row at a given concentration. If they were incorrect at least once, the 3‐AFC test was repeated using the next highest concentration of safflower oil. Based on previous studies (Stewart et al., 2010, 2011), it was expected that some participants would fail to identify the Oleic+ sample even at the highest oleic acid concentration (16.79 mM or 0.47% w/v). Such individuals were classified as fatty acid “insensitive” and effectively had no measurable threshold.

#### Perception of attributes in Oleic– and Oleic+ samples

To determine whether supplementation with oleic acid enhanced perception of fattiness and creaminess, participants evaluated five samples each of Oleic– and Oleic+ (sample # 2, 4, 6, 8, and 10 from Table 1), presented in a randomized order within each sample type. They used computerized ballots displaying a 100 mm labeled magnitude scale (LMS) anchored with the descriptors “barely detectable” and “strongest imaginable oral sensation” (Green et al., 1996) to rate the perceived intensity of fattiness, creaminess, sweetness, saltiness, sourness, and bitterness. A comments box was provided at the end of the ballot to collect any other verbal descriptions of the samples. All data were collected by FIZZ software.

### Data analyses

Individual and group detection thresholds were determined according to ASTM E1432‐04, where the threshold is defined as the level at which performance exceeds chance (50%). The group mean, and distribution of threshold responses were graphically depicted. Performance at each concentration was also calculated and plotted according to Antinone, Lawless, Ledford, and Johnston (1994) as an estimate of the discriminability of the samples. Threshold values were also examined as a function of CD36 genotype, gender, ethnicity and BMI. Differences in intensity ratings for the 6 attributes of Oleic– and Oleic+ samples were evaluated using repeated measures analysis of variance (ANOVA), with safflower oil concentration as the repeated measure. Main effects of CD36 genotype, gender, ethnicity, and BMI, as well as two‐way interactions, were examined in the ANOVA models. Analysis of covariance (ANCOVA) was also used to adjust for small, underlying differences due to gender or BMI; however, the findings matched those from the ANOVA models, so only the results from the ANOVA models are reported. Testing for normality was done with the Shapiro–Wilks test. Data were analyzed using Statistical Analysis Software (SAS version 9.4, The SAS Inst., Cary, NC, U.S.A.) using the general linear model (GLM) procedure, and planned, post hoc comparisons were made using Tukey's test following Bonferroni adjustment. Statistical significance was set at *P* ≤ 0.05 for all analyses.

## Results

### Participant characteristics

Participant characteristics are shown in Table 2. Sixty‐eight participants with a mean age of 25.3 ± 0.8 years and BMI of 24.0 ± 0.5 kg/m^2^ completed the study. The number of participants from each ethnic group was nearly identical (*n* = 36 Caucasian, *n* = 32 East Asian). Genotype distribution and allele frequencies for the CD36 rs1761667 SNP differed between Caucasians and East Asians (*χ* ^2^ = 6.35; *P* = 0.04 for genotype, and *χ* ^2^ = 6.64; *P* = 0.03 for allele frequencies by Fisher's Exact Test). The distribution of A and G allele frequencies for the CD36 rs1761667 SNP matched those for European and East Asian populations reported in 1000 Genomes (dbSNP Short Genetic Variations, 2017).

**Table 2:** Subject characteristics.^a^

	Caucasians (*n* = 36)	East Asians (*n* = 32)
Gender (*n*)		
Female	25	24
Male	11	8
Age (years)	25.3 ± 0.8	25.0 ± 0.9
BMI (kg/m^2^)	25.5 ± 0.7	22.3 ± 0.5
*CD36* rs1761667 genotype (*n*)^b^		
AA	8	5
AG	21	11
GG	7	16
Allele frequency (proportion)^b^		
A	0.51	0.32
G	0.49	0.67

^a^Values are means (±SEM) except as otherwise noted.

^b^Genotype distributions and allele frequencies differed between Caucasians and East Asians (*P* < 0.05).

### Oleic acid thresholds

**Figure 1:**
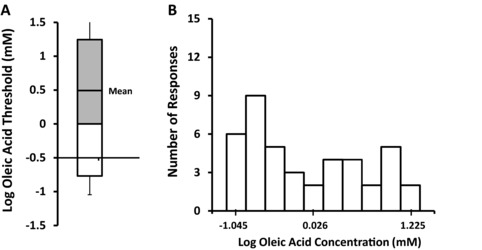
(A) Box plot showing the mean threshold detection of free oleic acid supplemented safflower oil emulsions. Sixty‐two percent of participants (*n* = 42) distinguished samples with added oleic acid (Oleic+) from samples without added oleic acid (Oleic–) in a 3‐Alternate Forced Choice test. The mean detection threshold for oleic acid in these “sensitive” participants was 2.9 ± 0.7 mM (0.08% w/v) oleic acid in a 2.81% safflower oil emulsion. (B) Histogram showing the distribution of threshold responses across oleic acid concentrations in “sensitive” individuals. Data were log transformed for presentation.

**Figure 2:**
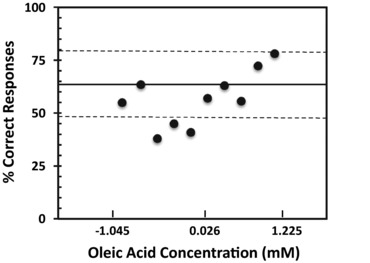
Percentage of correct responses in the 3‐AFC discrimination test at each oleic acid concentration. The solid line represents the 50% above chance performance for the “sensitive” participants (*n* = 42); the dashed lines represent the upper and lower bounds of the 95% confidence interval. Data were log transformed for presentation.

In the threshold test, 62% of participants (*n* = 42; 95% confidence interval, 49.7% to 74.3%; *P* < 0.05) were able to discriminate the Oleic+ from the Oleic– samples at greater than chance (50%) performance; the remaining participants could not reliably identify the Oleic+ sample at any concentration. The mean detection threshold for these “sensitive” individuals was 2.9 ± 0.7 mM (0.08% w/v) oleic acid in a 2.8% safflower oil emulsion. As shown in Figure 1, detection thresholds varied across a broad range of concentrations and the distribution of threshold responses was skewed, which is common for threshold data (ASTM E1432‐04). Figure 2 shows threshold performance plotted across concentrations. Although the percentage of correct judgments varied over the concentration range tested, most of the points lie within the confidence interval for the group. There was no effect of CD36 genotype, ethnic group, gender, age, or BMI on the ability to discriminate between Oleic+ and Oleic– samples (data not shown).

### Perception of fattiness and creaminess from Oleic– and Oleic+ samples

**Figure 3:**
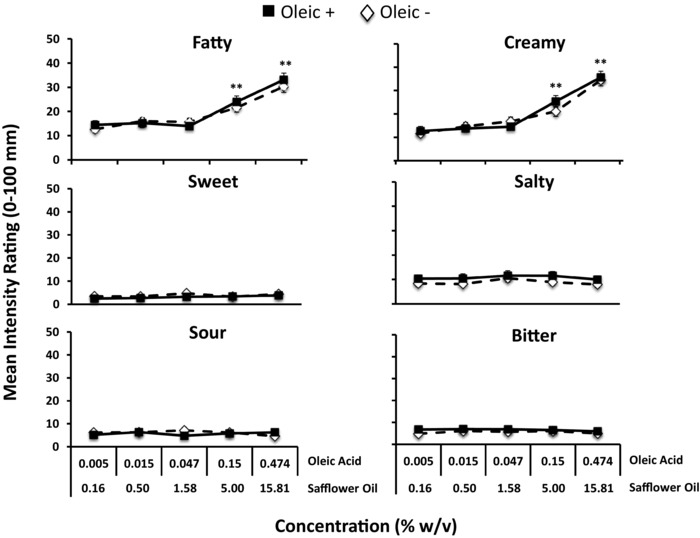
Perceived intensity of sensory attributes for safflower oil emulsions with or without added oleic acid in all participants. Oleic acid supplementation (Oleic+) did not enhance the perceived intensity of any of the sensory attributes compared with un‐supplemented (Oleic–) safflower oil emulsions. Oleic+ samples were prepared by adding free oleic acid at a constant ratio (3% w/v) of safflower oil. Perceived fattiness and creaminess rose in a similar manner with increasing safflower oil concentration in both Oleic– and Oleic+ samples at safflower oil concentrations exceeding 1.68%. ^**^Significantly different from 1.68% safflower oil (*P* < 0.001).

Figure 3 shows the intensity ratings for all attributes of the emulsions with increasing safflower oil concentrations in Oleic– and Oleic+ samples. Ratings for fattiness and creaminess did not rise until the safflower oil concentration exceeded 1.58% (w/v). Thereafter, ratings for fattiness and creaminess increased with increasing oil concentration (range = *P* < 0.01 to 0.001). Ratings for the side tastes (sweet, salty, sour, and bitter) did not change with increasing safflower oil concentrations and never exceeded a mean rating of 11.5 mm (less than “weak”) on the LMS. Importantly, supplementation with oleic acid did not enhance perception of fattiness or creaminess in the safflower emulsions (see Figure 3). There were no differences in fattiness or creaminess perception between Oleic+ and Oleic– samples at any concentration. Further, no differences in perception were found between participants who were previously identified as “sensitive” or “insensitive” in the threshold test (data not shown).

#### Effects of CD36 (rs1761667) genotype and ethnicity

**Figure 4:**
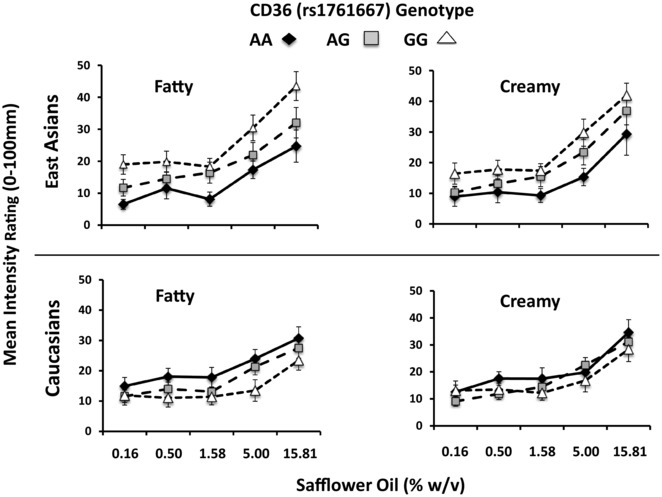
Perceived intensity of fatty and creamy attributes in safflower oil emulsions by CD36 rs1761667 genotype in East Asians (top panels) and Caucasians (bottom panels). Data shown are for Oleic– and Oleic+ samples combined. There was no effect of CD36 genotypes across concentrations.

Since oleic acid supplementation failed to enhance fat‐related attributes, the intensity ratings for Oleic– and Oleic+ samples were combined for all subsequent analyses and are presented as such in the figures. There were no main effects of CD36 genotype, ethnicity or their interaction on fattiness or creaminess ratings across the safflower oil concentrations (see Figure S1 and S2, respectively for main effects; *P* < 0.30 for all). Due to the high frequency of polymorphisms in East Asians (Elbers et al., 2012) and to examine potential differences in the directionality of the gene effects in our two subject groups, we also analyzed the data separately by ethnicity. Results showed that among East Asians, ratings of fattiness and creaminess tended to increase more rapidly across safflower oil concentrations in carriers of the GG genotype as compared to carriers of the AA genotype (see Figure 4). However, planned, post‐hoc comparisons failed to detect differences among the genotype groups at specific concentrations. In contrast, no differences were observed by CD36 genotype among the Caucasians (Figure 4).

**Figure 5:**
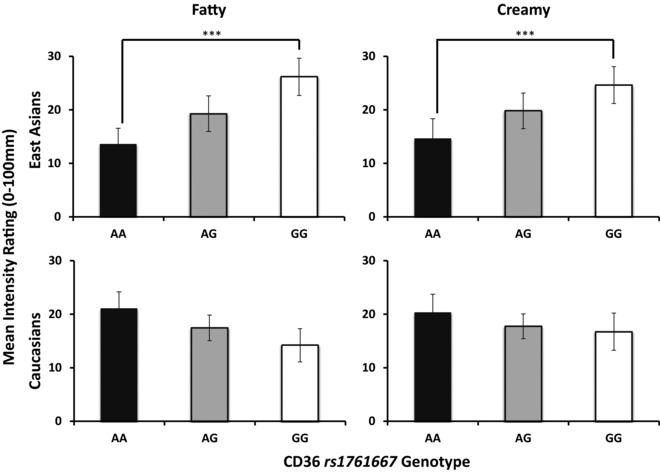
Perceived intensity of fatty and creamy attributes in safflower oil emulsions by CD36 rs1761667 genotype in East Asians (top panels) and Caucasians (bottom panels). Data shown were collapsed across all safflower oil concentrations. East Asians with the GG genotype gave higher intensity ratings for fatty and creamy attributes than East Asians with the AA genotype. No differences were detected among Caucasians. ^***^ *P* < 0.0001.

Figure 5 shows the main effect of CD36 genotype for the two ethnicities with the data collapsed across all concentrations. Overall, East Asians with the GG genotype perceived more fattiness and creaminess from the emulsions than their AA counterparts (*P* < 0.001 and *P* < 0.0001, respectively). Caucasians gave similar ratings for fattiness and creaminess, regardless of CD36 genotype.

## Discussion

This study contributes two novel findings to the literature. First, results showed that a majority of naïve, human assessors can detect oleic acid added to safflower oil emulsions at concentrations naturally present in edible commercial oils. Second, oral perception of fat was found to vary by CD36 genotype between Caucasians and East Asians.

With regard to oral detection, analyses revealed that nearly two‐thirds of our cohort exhibited a mean detection threshold of 2.9 ± 0.7 mM (0.08% w/v) oleic acid in a 2.8% safflower oil emulsion, which falls within the range of findings reported earlier using mineral oil (Chale‐Rush et al., 2007a, 2007b) or nonfat milk (Heinze et al., 2017; Stewart et al., 2010, 2011) as the vehicle. A sizable number (38%) of participants could not detect oleic acid within the tested range, consistent with previous reports showing large variation in detection of fatty acids in a liquid medium (Chale‐Rush et al., 2007a, 2007b; Stewart et al., 2011). Indeed, Stewart et al. (2010) observed that 51% of their participants were “insensitive” to oleic acid added to a milk base. Notably, previous studies (Running & Mattes, 2014; Tucker & Mattes, 2013) indicate that repeated testing improves the ability to detect fatty acids, suggesting that participants in our study deemed “insensitive” might acquire measurable thresholds with increased exposure.

In a recent study using milk‐based samples, Heinze et al. (2017) found that their cohort as a whole exhibited a similar threshold to that of our “sensitive” participants; they detected 5.57% canola oil spiked with a constant amount (3.80 mM) of oleic acid, and the threshold was substantially lower for the “hypersensitive” participants who detected the canola + oleic acid spiked samples at ∼4% canola oil. Why one‐third of our cohort failed to detect the oleic acid is unknown. It is possible that these “insensitive” participants found the samples to be perceptually challenging because the concentration of oleic acid was not fixed as in previous studies (Heinze et al., 2017; Stewart et al., 2010), but increased proportionally to the amount of safflower oil.

Contrary to our expectations, the addition of oleic acid to the emulsions did not heighten the intensity of fattiness and creaminess. A recent investigation evaluating the influence of fatty acid supplementation on hedonic reactions to chocolate came to a similar conclusion (Running, Hayes, & Ziegler, 2017). In that study, Running et al. (2017) reported that oleic acid supplementation had a modest impact on reducing liking, whereas, linoleic acid led to a strong rejection of the samples. This finding is consistent with previous research showing that linoleic acid is sensed as “fatty” or “scratchy,” depending on the concentration used (Galindo et al., 2012), and its threshold is 5.6 times lower than that of oleic acid (Running & Mattes, 2014). On the whole, these discoveries indicate that fatty acids exhibit distinct orosensory profiles that impact overall perception and food acceptability. Future investigations are needed to examine how supplementation using other long chain fatty acids might impact perception of fat‐associated attributes, as well as identify the concentration necessary to evoke a fatty sensation without causing irritation.

It is noteworthy that ratings for fattiness and creaminess remained flat across the lower concentrations of oil, not rising until safflower oil content reached 1.58% w/v. We did not measure physical properties of our samples such as variation in droplet size, droplet aggregation, viscosity, and lubricity (slipperiness) which can subtly influence textural sensations (Running & Mattes, 2014). van Aken, Vingerhoeds, and de Wijk (2011) studied the perception of fat‐related attributes in oil emulsions that were designed with different viscosities. Results showed that increasing viscosity had relatively little impact on fattiness and creaminess perception, but the attribute, mouthcoating was strongly related to fattiness and creaminess intensity. Returning to our data, it seems plausible that when safflower oil content exceeded 1.58% w/v, participants were able to discern oral cues related to mouthcoating/slipperiness that contributed to increasing fattiness and creaminess intensity. This observation deserves further investigation to better understand the in‐mouth perception of emulsified fats in food products.

We observed no main effect of CD36 genotype on oral detection of fatty acids and no interaction between CD36 rs1761667 genotype and ethnicity on threshold detection. In this respect, our findings differ from previous reports in the literature, which have repeatedly shown an effect of this SNP on fatty acid detection (Karmous et al., 2017; Melis et al., 2015; Mrizak et al., 2015; Pepino et al., 2012). However, the frequency of CD36 polymorphisms varies widely across ethnic group, especially in African Americans and East Asians (Elbers et al., 2012). Therefore, combined testing of Caucasians and East Asians in this study may have masked main effects of CD36 on fat perception. When we analyzed East Asians separately from Caucasians, we found that East Asians who had the GG genotype gave higher fattiness and creaminess ratings to the samples compared to those who had the AA genotype. No effect of this SNP was observed on the perception of fat‐associated attributes among Caucasian participants. Heightened ratings of fat perception among East Asians who were GG carriers may be due to increased CD36 protein expression (Love‐Gregory et al., 2011), although it is presently unclear why the same relationship was not observed in GG Caucasians in this study.

Our observation of a CD36 gene effect on the attribute ratings but not on threshold discrimination is not surprising since threshold acuity may not be strongly related to suprathreshold (that is, above threshold) taste intensity (Bartoshuk, 1978; Webb, Bolhuis, Cicerale, Hayes, & Keast, 2015). However, prior studies on CD36 genotypes and fat perception have not compared these two measures of perceptual ability. This gap in knowledge deserves additional study.

To our knowledge, only 2 investigations have examined the role of CD36 SNPs in the perception of fat‐containing foods. Studying an African American cohort with obesity, Keller et al. (2012) reported that carriers of the AA genotype of the rs1761667 SNP perceived greater creaminess from salad dressings independent of oil concentration, and showed greater liking for high‐fat foods compared to carriers of the GG genotype. In African Americans, A is the minor allele, while in Caucasians and Asians, G is the minor allele. These differences in minor allele frequency (MAF) across ethnic group may in part explain differences in genotype‐phenotype relationship reported across studies. Additionally, in the study by Keller et al. (2012), those with the CT or TT genotypes of the rs1527483 SNP in CD36 gave higher overall ratings for fat content in the samples. When Ong et al. (2017) examined the rs1527483 SNP in a Malaysian population of predominantly Chinese descent, they found similarly that participants who were homozygous for the T allele perceived more fat in both regular and reduced‐fat cream crackers. However, no robust relationships were observed between the rs1761667 SNP and fat perception in this latter investigation. Collectively, these findings demonstrate that CD36 polymorphisms may influence different features of fat perception, and effects of this gene may vary across ethnic groups. Comprehensive multiethnic studies are necessary to better characterize these effects.

This study had strengths and limitations. As strength, our participants did not perceive noticeable side tastes (for example, sweet, salty, sour, or bitter) from the samples, nor did they mention unpleasant oral sensations (for example, tingling or burning) in the comments box. These results support our findings of only trace amounts of oxidation products in the samples. As a limitation, we did not assess individual variation in lingual lipase activity (Kulkarni & Mattes, 2014) that could influence the availability of fatty acids to interact with oral fat receptors to enhance fat perception. Additionally, the small cohort size may have resulted in an amplification of the effects of the rs1761667 SNP; albeit, the MAFs observed in our Caucasian participants agree with population norms (dnSNP Short Genetic Variations, 2017) and values reported by Ong et al. (2017) for Malaysians of Chinese descent. Small sample sizes in several of the genotype groups strongly justifies repeating this study in a larger cohort to verify the current findings.

## Conclusions

This study demonstrated for the first time that humans can detect oleic acid when added to an oil‐in‐water emulsion at concentrations that may be present in commercial oils. However, supplementation with oleic acid at 3% w/v of the safflower oil did not enhance perceived fattiness or creaminess of the samples, at least under the conditions employed here. Thus, fatty acid supplementation may not be a successful strategy for enhancing the creaminess of foods such as salad dressings, mayonnaise and fluid dairy products. We also found that the rs1761667 SNP of CD36 associated with differences in the perception of fattiness and creaminess among East Asians, but not Caucasians. Obtaining a better understanding of the role of CD36 and other genes involved in oral fat detection and perception will provide insight towards the development of foods that meet the expectations of diverse consumers.

## Author Contributions

BJT and KLK conceived and designed the study; BJT directed the study; MM and MD conducted the study; KMS directed the lipid analyses; BB, MM, and KS analyzed the data; BB and BJT wrote the manuscript; KLK, KMS, MM and ITB critically evaluated the manuscript; all authors approved the final version.

## Conflicts of Interest

The authors disclose no conflicts of interest.

## Supporting information


**Figure S1**. Perceived intensity of fatty and creamy attributes in safflower oil emulsions with or without added oleic acid in East Asians.
**Figure S2**. Perceived intensity of fatty and creamy attributes in safflower oil emulsions with or without added oleic acid in Caucasians.
