# Supplementary material for: Effects of CD36 Genotype on Oral Perception of Oleic Acid Supplemented Safflower Oil Emulsions in Two Ethnic Groups: A Preliminary Study
Source: J Food Sci. 2018 Apr 16;83(5):1373–80. doi: 10.1111/1750-3841.14115 (PMC5969292; doi:10.1111/1750-3841.14115)
Supplement: Supplementary file 1 — Figure S1. Perceived intensity of fatty and creamy attributes in safflower oil emulsions with or without added oleic acid in East Asians. Figure S2. Perceived intensity of fatty and creamy attributes in safflower oil emulsions with or without added oleic acid in Caucasians. [file JFDS-83-1373-s001.pdf]

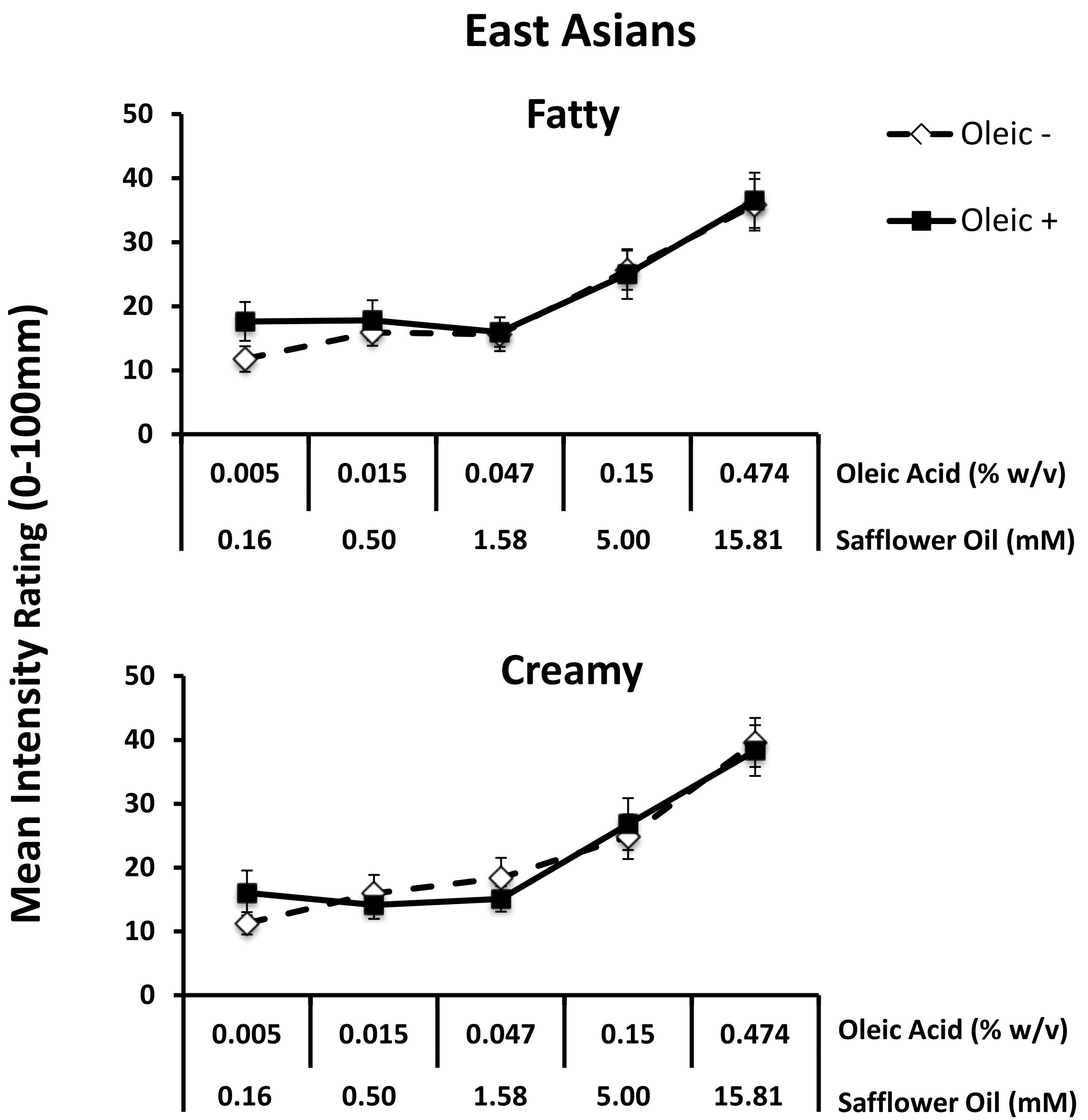

Supplemental Figure 1: Perceived intensity of fatty and creamy attributes in safflower oil emulsions with or without added oleic acid **in EAST ASIANS**. Oleic acid supplementation did not enhance the perception of these attributes across concentrations

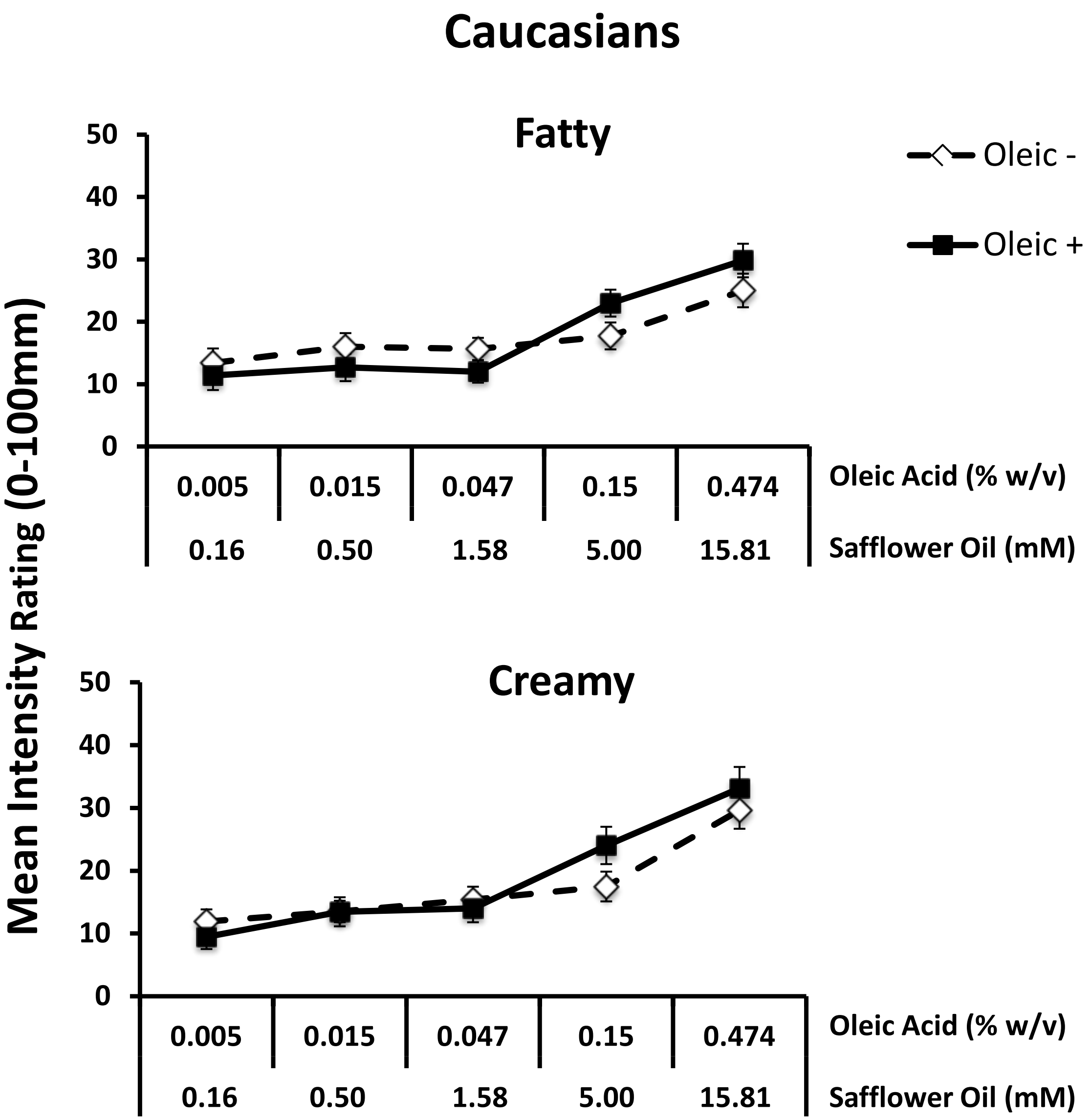

Supplemental Figure 2: Perceived intensity of fatty and creamy attributes in safflower oil emulsions with or without added oleic acid in **CAUCASIANS**. Oleic acid supplementation did not enhance the perception of these attributes across concentrations.
